# An exploration of values among consumers seeking treatment for borderline personality disorder

**DOI:** 10.1186/s40479-018-0085-9

**Published:** 2018-04-15

**Authors:** Simone R. Mohi, Frank P. Deane, Anne Bailey, Dianne Mooney-Reh, Danielle Ciaglia

**Affiliations:** 1 School of Psychology, University of Wollongong, Office: 22.G15, Wollongong, NSW 2522 Australia; 2 Illawarra Institute for Mental Health, School of Psychology, University of Wollongong, Office: 22.G18, Wollongong, NSW 2522 Australia; 3 Illawarra Shoalhaven Local Health District, Wollongong, NSW Australia

**Keywords:** Borderline personality disorder, Treatment, Values, Life domains

## Abstract

**Background:**

Consumer feedback identifies a new challenge in the treatment of borderline personality disorder (BPD) is to address the discrepancy between clinical treatment targets and the more personally meaningful goals people are seeking in treatment. This highlights the need to increase clarification of people’s values and link these to therapy goals. The current study explores ways in which individuals with BPD identify with values across key life domains.

**Methods:**

At initial assessment 106 consumer participants attending an outpatient clinic for the treatment of BPD completed the Personal Values Questionnaire by Blackledge and colleagues. This 90-item measure asks participants to respond to different value appraisals such as importance, commitment, desire to improve, success and, motivation across nine life domains. These included: relationships, health & wellbeing, education & personal development, work & career, spirituality, recreation & leisure, and community involvement.

**Results:**

A consistent pattern of value appraisals was found across all life domains. Specifically, life domains were endorsed as highly important but participants reported significantly lower levels of value commitment, desire to improve and success. Successful value pursuit also related differentially to value motivations (internal vs. external) depending on the particular life domain. Relationships with family, friends and romantic partners, as well as health & wellbeing were most important compared to other life domains.

**Conclusions:**

The findings suggest that people with BPD identify with values and prioritise different life domains in terms of importance. Our results show discrepancies between higher importance and lower commitment, desire to improve and success at living in line with what is valued. Identification of such discrepancies provides opportunities to more effectively support consumers with BPD to prioritise goals that are consistent with valued domains. These findings offer new insights for cultivating the personal meaning consumers are currently seeking in BPD treatments.

## Background

Considerable progress in the treatment of Borderline Personality Disorder (BPD) has been made in the last two decades however, accumulating consumer feedback identifies a new challenge is to address the discrepancy between mainstream clinical targets and the personal recovery goals people have in treatment [1–5]. It has become clear that the improvements monitored and measured in the clinical literature, such as symptom reduction, does not necessarily correspond with people’s actual evaluations of meaningful progress and life improvement on the road to recovery [6, 7]. Initial insight into the consumer experience specific to BPD treatments comes from Katsakou and colleagues [2] who conducted a series of qualitative interviews across a sample of 48 service users from different mental health settings. These authors systematically investigated consumer perspectives on personal recovery in the treatment of BPD. Thematic content analysis identified a common theme in consumer feedback was a need to help people make personal meaning in their lives and progress toward their personal goals [2]. The authors concluded that while specialist therapies for BPD are addressing important treatment targets, such as reductions in self-harm, the more personally meaningful goals consumers are seeking warrant more attention in treatment.

Similar conclusions can be drawn from a recent meta-synthesis of qualitative studies highlighting treatment characteristics valued by consumers [3] as well as a recent meta- analytic systematic review of longitudinal recovery data highlighting the need for a broader recovery vision in the treatment of BPD [5]. Ng, Bourke, and Grenyer [5] analysed 19 studies, 11 unique cohorts comprising 1122 BPD treatment consumers. They concluded that there is a need for treatment providers to advance broader recovery improvements for consumers across different life domains such as engaging in meaningful work, having satisfying relationships and living a contributing life. These findings highlight the need to increase clarification of people’s values and link these to personally meaningful goals in the treatment of BPD. However, research that assesses values in people with BPD is needed in order to inform the development of values-focussed interventions.

Seminal cross cultural research into values conducted by Schwartz [8] drew attention to that fact that values are a universal source of motivation and fundamental driver of behaviour for human beings across cultures. While values have been conceptualized in a number of ways in psychology [8–10] they can be generally understood as guiding life principles that provide a basis from which people devise their personally meaningful goals and therefore influence daily decision-making [11]. The empirical measurement and ways in which values have been operationalised across different research studies has varied depending on the assessment measure used. For example, both the Valued Living Questionnaire (VLQ [12]) and Personal Values Questionnaire (PVQ [13]) operationalise values by organising them into life domains that are typically valued by people, such as relationships, health, career and education etc. This is distinctly different to Schwartz’s Value Survey (SVS) which examines qualitatively different aspects of people’s values according to Schwartz’s theory on values [8]. For example, the SVS assesses the importance people ascribe to value items as life-guiding principles such as achievement (success, capability, ambition, influence on people and events) and benevolence (helpfulness, honesty, forgiveness, loyalty, responsibility).

A recent empirical study utilising the VLQ by Huguelet and colleagues [14] investigated whether values may be determinants of life meaning and fulfilment among 176 patients with a variety of long standing psychiatric disorders. It was found that the presence and enactment of values among patients was a valid determinant of life meaning including a person’s sense of fulfilment and life goals which was a determinant of symptomology. The authors concluded that life meaning relies on values and that a lack of meaning in life may foster symptomology. It was argued that clinical interventions need to focus more on values in helping patients to make meaning in their life. The cross sectional nature of this latter study weakens causal interpretation however a prospective longitudinal study found increases in purpose and meaning in life preceded improvements in mental health after controlling for a range of potentially confounding variables [15].

Clinical scholars and practicing clinicians from various theoretical orientations have argued that including a values based approach in the treatment of BPD will translate into gains for consumers and treatment teams by establishing conditions that make therapy more effective [14, 16–18]. Cameron and colleagues [17] refer to the ongoing challenges to BPD treatments such as low levels of motivation and engagement as well as high dropout rates. Studies examining dropout rates of individuals diagnosed with BPD in Dialectal Behaviour Therapy (DBT) range from 22% [19] to 46% [20] and up to 52% in outpatient settings [21]. One study found that treatment non-completers had significantly higher rates of rehospitalisation (22%) compared to treatment completers (11%) at 1 year follow-up [22]. The implications of these statistics is what prompted the development of clinical recommendations advocating for the integration of a values-based approach into mainstream BPD treatments [17].Value focused interventions may also have therapeutic potential for targeting some of the core features of borderline pathology including, identity disturbance and chronic feelings of emptiness [23, 24] since connection to one’s values can provide purposeful direction and meaning in life [14]. It has long been recognized that a connection to one’s values is essential to the development of a person’s sense of self, identity and personal fulfilment [10].

Helping people to identify, connect to and operationalise their personal values across key life domain areas, such as relationships, career, well-being and health, is a core component of Acceptance and Commitment Therapy (ACT [11]). Meta-analytic reviews suggest that ACT is an effective treatment across a wide variety of clinical populations [25]. Values focused interventions are theorised to be an important part of ACT but component analyses are yet to validate the unique contribution of these value-focused interventions. To date we have found only one published study that has trialled a values focused intervention for BPD treatment consumers [18]. This pilot study by Morton and colleagues [18] trialled a group based intervention delivered as two-hour weekly sessions over 12 weeks. Consumer participants were randomly assigned to either treatment-as-usual plus the values intervention (ACT+TAU; *N* = 21) or treatment-as-usual (TAU; *N* = 20). There was significantly more improvement from baseline for the ACT+TAU condition than the TAU on all primary outcome variables measured, including: self-rated BPD symptoms, feelings of hopelessness, psychological flexibility, emotion regulation skills, mindfulness, and fear of emotions. These results provide important preliminary evidence for the potential benefits of value based interventions in the treatment of BPD however a limitation of the study was that a measure of values was not included. There is a need for more research to better understand the importance of different value domains amongst people with BPD seeking treatment.

According to Self-Determination Theory (SDT) people are more successful in pursuing their values when they feel motivated towards them [26, 27]. However, not all motivational sources are assumed equal when it comes to predicting successful value pursuit across different life domains. A key theoretical distinction made by SDT is that between internalised and externalised motivation sources. SDT assumes that internally sourced motivation is the stronger predictor of value success because the pursuit of related goals are experienced as personally important and self-regulated where inherent desire to perform tasks comes from within the person [26, 28]. This is in contrast to externally sourced motivation which is less predictive of value-based success because it is experienced as more imposed and driven by external rewards and/or avoidance of punishment where the desire to perform related tasks is experienced as controlled by an outside source [29]. Despite the fact that these latter theoretical predictions have been empirically substantiated in a number of population samples [29–33] it is not yet known whether they extend to a BPD population. Research into what motivates people with BPD to pursue values in key life areas could usefully inform treatment providers better positioning them to work with the motivational needs clients have in service of pursing what is important to them.

### Current study

The primary aim of the current study was to describe the ways in which individuals with BPD who are seeking treatment identify with values across a number of key life domains as assessed by the Personal Values Questionnaire [13]. Specifically, the study aims to explore value importance, commitment, desire to improve and perceived success across a number of life domains (e.g., relationships, health & wellbeing and work & career). In addition, explore the relationship between different motivational sources and reported value success across life domains. Based on predictions derived from SDT, we hypothesized that those values held for more internalised motivations would be more strongly related to value success than those that are more externally motivated.

## Method

### Participants

Participants in this study were 106 adults with a diagnosis of BPD who were referred to the Illawarra Affect Regulation Clinic (ARC) for the assessment and treatment of BPD. ARC provides a yearlong BPD treatment program to local community members and is operated as a collaborative enterprise between the at the University of Wollongong Clinical Psychology training clinic and the Specialist Psychological Service at Illawarra Community Mental Health in New South Wales, Australia. The treatment program is integrative and based on evidence based practice. Hence, Dialectical Behaviour Therapy (DBT) forms one of the cornerstones of the program, although other clinical treatment interventions are integrated including the trial of a values-focused intervention. Consumer referrals to ARC are made up of inpatient and community, public and private, government and non-government institutions in the Illawarra area. ARC has been in operation for over a decade during which time data collection for a larger study has occurred, hence a research component of ARC is evaluating the treatment service it provides.

For the current study, participants included those who met five or more BPD criteria on the Structured Clinical Interview for the Diagnostic and Statistical Manual of Disorders, Fourth Edition (DSM-IV [34]) which was administered by both clinical psychologists and clinical psychologist interns. Participants who met the DSM-IV criteria disorder categories schizophrenia and other psychotic disorders or pervasive developmental disorders were excluded from participation in this study. Participation in the study was voluntary and those who self-selected to participate signed the informed consent form prior to commencement. All participants were Australian adult citizens comprising 101 females and 5 males. Mean age for the sample was 29.92 years (*SD* = 10.29; range 18 to 60). Mean years of = education was 11.40 (*SD* = 1.96; range 7 to 21). Relationship status was 55.7% single, 38.7% were in a relationship (i.e. married, de facto, partner, fiancé) and 5.7% single or divorced. Data obtained from 5 participants were too incomplete to be used and therefore was excluded from the analyses.

### Measures

The following values assessment measure was administered to participants following the initial diagnostic assessment interview or prior to the commencement of ARC’s treatment program.

The Personal Values Questionnaire (PVQ [21]). The PVQ was originally adapted from a well-psychometrically established assessment measure of personal strivings developed by Sheldon and colleagues [23–25]. The PVQ itself is a 90-item self-report questionnaire which has been used to assess people’s values across key life areas in a number of different population samples and prior evidence suggests sound criterion-related validity [26–29]. The PVQ describes values as in life domains (e.g. *‘Personal Value 1: Family Relationships’, ‘Personal Value 2: Friendships’*) and participants are instructed to write down what they value within each of the nine respective life domains assessed including: 1) family relationships, 2) friendships/social relationships, 3) couples/romantic relationships, 4) work & career, 5) education, personal growth & development, 6) recreation activity, leisure & sport, 7) spirituality & religion, 8) community involvement & citizenship and, 9) health & well-being. Noteworthy is that only a small percentage of the sample responded to the written section of the PVQ and for this reason qualitative data analysis was not conducted.

The quantitative aspect of the measure instructs participants to rate value appraisals and value motivations for each life domain of personal relevance on a 5-point Likert scale. Value appraisals measured include value commitment, value importance, desire to improve in this value and, value success. For example, for each life domain participants were asked to rate *‘How important is this value to you?’* on a scale of 1 (*not at all important*) to 5 (*extremely important*). For rating value success participants were asked: *‘In the last 10 weeks, I have been this successful in living this value’*. With regard to assessing different value motivations, including both intrinsic and extrinsic motivations, participants are asked to rate the extent to which they pursue each personally relevant value in each life domain for different motives on a 5-point Likert scale, 1 (*not at all*) to 5 (*entirely*). Specifically, participants were asked to rate the extent that they held a particular value for externalized/social reasons (e.g. *‘I value this because somebody else wants me to’, ‘I value this because I would feel ashamed, guilty, or anxious if I didn't’*) vs internalized reasons (e.g. *‘These values are important to me, whether or not others agree’, ‘Living consistently with these values makes my life more meaningful’, ‘I experience fun and enjoyment when I live consistently with these values’*). In this research, we were specifically interested in understanding value success and different motivational sources as described by both Self Determination Theory (STD) and recent research on values and motivation. In line with the work of Jambrak and colleagues [31], we analysed four types of motivation separately to see it’s alignment with values success. Specifically, for each participant, a total of four motivation scores were calculated. In line with previous SDT research [29, 31] we used an aggregated intrinsic motivation score (AGMS) calculated by subtracting the total external motivation score from the total internal motivation. The intrinsic motivation score (IMS) and extrinsic motivation score (EMS) were calculated by averaging the intrinsic items and extrinsic items respectively. An additive motivation score (ADMS) was calculated by taking an average of combining both extrinsic and intrinsic items.

### Procedure

Approval for the current study was obtained from the Human Research Ethics Committee of the University of Wollongong. Assessment measures for the current study were administered to participants following their initial diagnostic assessment interview or prior to the commencement of treatment at ARC. Participants were informed of the current study at that time and were provided with information in both verbal and written forms outlining the aims of the research. Participation in the study was voluntary and those who self-selected to participate signed the informed consent form prior to commencement.

### Data analysis

Data were analysed using SPSS 21.0 for Windows. There were nine life domains measured and as participants were asked to respond to personally relevant values, not everyone responded to all life domains. As a result the missing data items were managed by listwise deletion a decision made based on our statistical analytical approach with the aim to determine potential differences between the life domains and value appraisals. Some variables within the nine life domains were skewed so non-parametric tests were conducted. In order to determine whether there were overall differences in the four value appraisal ratings they were collapsed across the nine life domains with mean appraisal scores calculated by averaging ratings for value importance, value commitment, desire to improve and value success. A Friedman’s test was conducted between these overall value appraisal scores to determine any potential differences. These were followed by pairwise comparisons using Wilcoxon tests to determine the nature of those differences.

In order to determine any relative differences in the strength of value appraisals between the different life domains nonparametric Friedman’s tests (four) were conducted and then followed by post-hoc Wilcoxon tests. All tests were two-tailed with *p* < .05. No adjustment to the *p*-value was made to control for multiple comparisons given the relatively exploratory nature of the research. Finally, to assess the magnitude and direction of the relationship between value motivations (e.g. internal and external) and value success for each life domain, Spearman’s rank correlation coefficients (*ρ*) were calculated.

## Results

### Pattern of value appraisals: Importance, commitment, desire to improve, and success

A Friedman test was conducted to test the overall differences in the four value appraisal ratings (collapsed across the nine life domains). The results of the Friedman test indicated significantly different means between value appraisals, χ^2^ (3, *N* = 62) = 125.975, *p* < .001). Pairwise comparisons using Wilcoxon tests indicated that level of value importance was significantly higher than all other value appraisals including value commitment, desire to improve and value success. Further, value commitment and desire to improve were not significantly different. And finally, value success was significantly lower than all other value appraisals. Thus the overall pattern of value appraisals across all life domains was: Importance > Desire to Improve = Commitment > Success. These results suggest that although life domains are identified as being relatively important, people’s rated levels of commitment, desire to improve, and success are lower. Table 1 shows descriptive statistics for the four value appraisals of importance, commitment, desire to improve and success for each of the nine life domains measured.

**Table 1:** Life domain by value appraisal. Descriptives: mean rank, mean, 95% Confidence Interval (CI) (Listwise by column)

	Importance (*n* = 68)			Commitment (*n* = 68)			Desire to improve (*n* = 68)			Success (*n* = 66)		
	Rank	*M*	95% CI	Rank	*M*	95% CI	Rank	*M*	95% CI	Rank	*M*	95% CI
Romantic relationships	5.89 ^a^	4.56	4.35–4.78	5.67 ^a^	3.93	3.63–4.23	5.69 ^ab^	3.95	3.64–4.27	5.18^abc^	2.70	2.09–2.75
Family relationships	5.65 ^a^	4.54	4.37–4.72	5.38 ^ac^	3.79	3.53–4.06	5.18 ^bc^	3.71	3.43–3.99	5.39^ab^	2.61	2.31–2.90
Friendships	5.41^a^	4.51	4.37–4.66	5.86 ^a^	3.96	3.70–4.21	4.33 ^cef^	3.39	3.08–3.71	5.88^a^	2.70	2.42–2.96
Health & wellbeing	5.32 ^ab^	4.46	4.27–4.64	4.65 ^bcde^	3.44	3.13–3.75	6.05 ^a^	4.12	3.83–4.41	5.04^abc^	2.39	2.09–2.69
Work & career	4.98 ^b^	4.31	4.07–4.54	5.21^ad^	3.74	3.45–4.02	4.98 ^bce^	3.64	3.30–3.96	4.53^bc^	2.27	1.93–2.61
Recreation & leisure	4.82^b^	4.25	4.02–4.48	4.32^be^	3.31	3.01–3.60	5.43 ^bd^	3.79	3.49–4.08	4.38^c^	2.17	1.87–2.46
Spirituality & religion	4.43^c^	3.97	3.67–4.27	4.99 ^ae^	3.53	3.19–3.86	4.60 ^bce^	3.56	3.23–3.88	5.28^abd^	2.62	2.31–2.93
Education & development	4.38^c^	4.07	3.81–4.33	4.43^be^	3.44	3.16–3.72	4.98 ^bce^	3.74	3.43–4.05	4.46^bc^	2.27	1.96–2.57
Community & citizenship	4.12^c^	3.84	3.52–4.16	4.47 ^be^	3.34	3.01–3.66	3.77 ^f^	3.03	2.70–3.56	4.87^abc^	2.41	2.05–2.76
Total *		4.23	4.10–4.35		3.61	3.43–3.78		3.64	3.44–3.84		2.45	2.26–2.63

Note. Mean scores ranged from 0 to 5; higher scores represent greater levels of value construct measured

a,b,c,d,e,f Mean ranks that differ from each other within columns at *p* < .01, do not share a letter. (20) For example, in the first column under the title ‘Importance’ it can be seen that romantic relationships and work & career domains do not share a letter in common, this means that they do significantly differ from one another. Conversely, in the same column it can be seen that romantic relationships, family relationships and friendships all share the letter ‘a’, this means that these three domains do not significantly differ from one another. * Wilks’ Lambda = 0.11, *F* (3, 59) = 153.34, *p* < .001

### Relative strength of value appraisal for each life domain

The relative differences in the strength of value appraisals were compared across life domains. Hence, to determine the relative strength of the four value appraisals of importance, commitment, desire to improve, and success for each life domain, non-parametric Friedman tests was performed within value appraisal across life domains. If the Friedman test was significant it was followed by a series of post-hoc Wilcoxon tests. Wilcoxon tests were two-tailed and a significance level of *p* < .05 was used for each comparison due to the exploratory nature of this research. Results for each value appraisal are summarised below and in Table 1.

#### Value importance

Significant results from the Friedman test indicated a difference in the level of importance between the different life domains: χ^2^ (8, *N* = 68) = 40.88, *p* < .001). Table 1 presents the results from the Wilcoxon comparisons tests which shows that relationships (i.e. family, friends and romantic) were rated as significantly more important than all other life domains with the exception of health and wellbeing which was perceived as equally important.

#### Value commitment

Significant results from the Friedman test indicated a difference in the level of commitment between the different life domains: χ^2^ (8, *N* = 68) = 29.07, *p* < .001). The Wilcoxon tests indicate that significantly higher levels of commitment were present for relationships (i.e. family, friends and romantic) compared to other life domains including education & personal development, recreation & leisure, and community involvement & citizenship life domains (see Table 1).

#### Desire to improve value

Significant results from the Friedman test indicated a difference in the level of desire to improve between the different life domains: χ^2^ (8, *N* = 68) = 29.07, *p* < .001). The Wilcoxon tests indicate that desire to improve was strongest for health & wellbeing, which was significantly higher than all other life domains, with the exception of romantic relationships (see Table 1).

#### Value success

Significant results from the Friedman test indicated a difference in the level of success between the different life domains, χ^2^ (8, *N* = 66) = 16.72, *p* < .001). The Wilcoxon tests showed that significantly higher levels of value success were reported for friendships compared to work and career, education, personal development as well as recreation and leisure. In addition, value success ratings for family relationships were higher than for recreation & leisure. Value success ratings did not vary significantly between all other domains (see Table 1). The greatest discrepancy between desire to improve and success was for health & wellbeing (mean scores: desire 4.12/5 > success 2.39/5).

### Motivation and value success in life domains

To assess the magnitude and direction of the relationship between the value motivations and success for each life domain, Spearman’s rank correlation coefficients (*ρ*) were calculated. Table 2 shows that both intrinsic and extrinsic motivations were significantly correlated with reported value success but differed depending on the life domain. The results showed that intrinsic motivation was significantly positively correlated with value success for four of the nine life domains including: friendships, work & career, spirituality & religion, community & citizenship. Three life domains were significantly positively correlated with extrinsic motivation including: romantic relationships, education and development and community & citizenship. Table 2 further indicates that for six of the nine life domains value success was related to the additive effects of both internal and external motivations. Of note is the finding that value success in family relationships was most strongly related to internal motivation with low levels of conflicting motivations (intrinsic minus extrinsic). In addition, the finding that value success for two of the life domains appeared to be unrelated to motivation. That is, value success in health & wellbeing as well as recreation & leisure were found to have no significant correlation to any motivation source.

**Table 2:** Value success and motivation. Non-Parametric correlations (Listwise by row)

Value success	n	Intrinsic Motivation	Extrinsic Motivation	Intrinsic minus Extrinsic	Intrinsic plus Extrinsic
Family relationships	101	.14	−.17	.22*	−.06
Friendships	100	.28**	.15	−.00	.21*
Romantic relationships	94	.19	.23*	−.08	.30**
Work & career	94	.23*	.19	−.01	.24*
Education & development	85	.15	.21*	−.03	.22*
Recreation & leisure	97	.12	.13	−.03	.16
Spirituality & religion	88	.35**	.13	.18	.30**
Community & citizenship	83	.64**	.34**	.19	.56**
Health & wellbeing	97	.13	.12	−.05	.14

Note: ^*^ *p* < .05 (2-tailed). ^**^ *p* < .01 level (1-tailed)

## Discussion

Our aim was to investigate the ways in which people seeking treatment for BPD identify with values across a number of key life domains including relationships, health & wellbeing, education & personal development, work & career, spirituality, recreation & leisure, and community involvement. We used the Personal Values Questionnaire [13] to investigate people’s experience of values in different life domains, and examined the ways in which people identify with different value appraisals, such as value importance, value commitment, desire to improve in values and value success. The results of our study revealed a consistently robust pattern of value appraisals across the different life domains assessed. We found that life domains were consistently endorsed as highly important to people however they reported relatively lower levels of value commitment, desire to improve and perceived success. In addition to this pattern of value appraisal responses, some life domains were viewed as being more important than others. People rated their personal relationships with family, friends and romantic partners, as well as their health & wellbeing to be most important when compared to all other life domains assessed including: work & career, education & personal development, leisure & recreation, spirituality, and community involvement. Noteworthy is that levels of personal commitment to values were also rated highest for relationships when compared to all other life domains and the greatest discrepancy between desire to improve and value success was found for health & wellbeing.

Taken together, these results have a number of implications. Firstly, our findings suggest that people with BPD identify that values associated with a number of key life domains are important to them. There were consistent discrepancies between how important a life domain was rated and significantly lower levels of value commitment, desire to improve and success. Awareness of these discrepancies may be helpful in supporting clients to prioritising individual treatment goals. For example, by helping individuals to clarify life domains that they value most and where they have a high desire to improve will help clarify their goals in treatment. The finding that people valued their health & wellbeing and personal relationships above all other life domains considered here suggests that increased attention to these life areas may be warranted in treatment.

Interpersonal relationships were most important in the sample. Problematic interpersonal relationships and functioning is inherent to borderline pathology [23, 24] and prominent treatment approaches target relational functioning through teaching relationship skills (e.g. in DBT) and developing mentalizing capacities for understanding states of self and others in context of relationship (e.g. in both Mentalization-based therapy and Transference-Focused Psychotherapy). In addition to these established methods a more structured values clarification process may help identify the importance of personal relationships as a treatment goal. Should such values-driven goals be identified then the link between this goal and the treatment focus on relationship skill development can be more explicitly linked.

The secondary aim of the study was to see whether different motivational sources (e.g. internal vs. external) influenced people’s success in values across different life domains. Drawing upon Self-Determination Theory [32] it was hypothesized that internalized motivations would be more strongly associated with higher levels of value success as opposed to those more externally motivated. The current results did not fully support this hypothesis. Contrary to predictions motivational sources (e.g. internal vs. external) related differentially to people’s reports of value success dependant on the life domain and each relationship was related to a different balance of both internal and external motivations. For most life domains value success was related to the additive effects of both internal and external motivations. These findings suggest that while there may be a predominance of one reward orientation over another in any given valued life domain it is common for both extrinsic and internal motivation to be relevant. Noteworthy is that family relationships and education domains were exceptions to the general finding that the additive effects of both internal and external motivations were related to value success. Reported success in family relationships was found to be most strongly related to internal motivation with low levels of conflicting motivations (intrinsic minus extrinsic). This means people are likely to engage in their family values more successfully for reasons experienced as inherently valuable to them and perceived success is likely to be lower when engagement in family values is driven by externally motivated reasons. Extrinsic motivations might include behaving out of a sense of duty and/or obligation, which seems to undermine that which is inherently valued. With regard to education & personal development, value success was found to be most strongly related to externally sourced motivation. This indicates that people are more likely to engage successfully in educational and personal development values for more externalized reasons, such as contingencies related to self-worth and/or social recognition. A final observation is the non-significant relationship between value success and motivation for two of the life domains assessed. Health & wellbeing and recreation & leisure were found to have no significant relationship with internal and/or external motivation sources. While this result may indicate value success for these two life domains is unrelated to any motivational source that seems unlikely given the robust pattern of results found between motivation and value success in the other life domains assessed here. Alternatively, this result may be more a function of the fact that overall motivation levels for health & wellbeing and recreation & leisure were particularly low. It may also be that individuals with BPD are particularly affected in these two life domains and they have more difficulty rating their internal and external motivation in these life areas.

The current results add to extensive empirical findings related to Self-Determination Theory [26–28, 31] and have implications for treatment providers when working with motivation and values in treatment. Firstly, the findings indicate that different motives can drive the pursuit of values dependent on the life domain. For example, in vocational domains, extrinsic motivation seems to play a larger role, while in relationships, intrinsic motivation is more strongly related to success. While there may be a predominance of one reward orientation over another in any given valued life domain, it is common for both extrinsic and internal motivation to be influential. Clinicians can make use of identifying and understanding the type of motives driving the pursuit of particular values with their clients and assist in harnessing motivations in the service of personally meaningful goals. For example, for some life domains tapping into external motivation may be necessary in the first instance to get people initially engaged in longer term goals which may then lead to increased internal motivation required to sustain longer term success [27, 28].

Administering values-based assessment approaches and/or utilizing motivational interviewing techniques are likely to be useful tools in this process [35, 36]. For example, treatment providers can assist people to connect with and operationalise their values by facilitating a step by step process initially utilising a structured values assessment measure [36]. Following the identification of values, strategies can be implemented to develop congruence between values and behaviour also known as committed action [37]. In support of this process, clients can be helped to set short and longer-term value-congruent goals, develop and implement action plans in service of those goals which can be monitored overtime [36].

### Limitations

Several limitations must be considered when interpreting the current study’s results. Firstly, the individuals who participated in the study were from a relatively limited geographical district from within New South Wales Australia all of whom participated on a voluntarily basis and therefore may have been more intrinsically motivated to participate than those who declined. The issue of potential sample bias could be addressed, in part, by future research that involves a broader recruitment approach to obtaining its sample. Secondly, the data analysed in this study was cross sectional and therefore the results obtained for the variables measured may be a function of time point. The focus of this study was predominantly descriptive and other measures to control for potential covariates (e.g. depression) were not included. There is a need for future research to explore the extent that other variables are related to or potentially influence values or motivations. For example, it is possible that higher levels of depression could hinder the desire to improve values related goals.

### Future directions

The extent to which we could assess people’s values was limited to the method of assessment utilised, namely the Personal Values Questionnaire [13] which operationalises values by organising them into life domains typically valued by people. Future research could usefully utilise an alternative assessment tool, such as Schwartz’s Value Survey (SVS [8]) which would extend upon our findings examining qualitatively different aspects of people’s values according to Schwartz’s theory on values [36]. For example, the SVS assesses the importance people ascribe to value items as life-guiding principles such as achievement (success, capability, ambition, influence on people and events) and benevolence (helpfulness, honesty, forgiveness, loyalty, responsibility). In addition, future research could usefully extend upon the original pilot study of Morton and colleagues [18] who trialled a values focused intervention for BPD treatment consumers. It would be useful to see whether inclusion of a values approach translates into the hypothesized therapeutic benefits such as increased treatment persistence, reductions in dropout and moderation of core features of borderline pathology such as identity disturbance and chronic feelings of emptiness through strengthening a sense of meaning and purpose.

## Conclusions

Prior research suggests that individuals with BPD want treatment to support them to meet a wider range of recovery outcomes, not just those specific to symptoms or behaviours associated with their diagnosis [1–5]. The current study indicates that when asked, people diagnosed with BPD who are seeking treatment are able to identify with values and prioritise different life domains in terms of importance. Close relationships with family, friends and romantic partners as well as one's health & wellbeing seem most important compared to other life domains. The findings reveal discrepancies between how important valued life domains are and lower levels of value commitment, desire to improve and success. Identification of values and discussing such discrepancies in treatment may be helpful in supporting clients to prioritise their individual goals. Perceived value success related differentially to value motivations (internal vs. external) depending on the particular life domain. By understanding what motivates people to pursue values in key life areas better positions treatment providers to work with the motivational needs clients have in service of pursing what is important to them. Therapeutic approaches such as ACT help people to identify, connect to and operationalise their values across key life areas. Broadening BPD treatments to include a focus on people’s values may be one way to help close the gap between treatment targets and the more personally meaningful goals people are wishing to pursue in treatment. Future research is needed to explore the relevance of values in relation to BPD symptomology. Then there is the potential to trial interventions that support values-focused change.

## Abbreviations

ACT: Acceptance and commitment therapy
ADMS: Additive motivation score
AGMS: Aggregated intrinsic motivation score
ARC: Affect regulation clinic
BPD: Borderline personality disorder
DBT: Dialectical behavior therapy
EMS: Extrinsic motivation score
IMS: Intrinsic motivation score
PVQ: Personal values questionnaire
SDT: Self-determination theory
TAU: Treatment-as-usual;
